# Alternative Splicing of Pre-mRNA in the Control of Immune Activity

**DOI:** 10.3390/genes12040574

**Published:** 2021-04-15

**Authors:** Zhongjing Su, Dongyang Huang

**Affiliations:** 1 Department of Histology and Embryology, Shantou University Medical College, No. 22, Xinling Road, Shantou 515041, China; 2 Department of Cell Biology, Shantou University Medical College, No. 22, Xinling Road, Shantou 515041, China

**Keywords:** alternative splicing, immune activity, non-coding RNA

## Abstract

The human immune response is a complex process that responds to numerous exogenous antigens in preventing infection by microorganisms, as well as to endogenous components in the surveillance of tumors and autoimmune diseases, and a great number of molecules are necessary to carry the functional complexity of immune activity. Alternative splicing of pre-mRNA plays an important role in immune cell development and regulation of immune activity through yielding diverse transcriptional isoforms to supplement the function of limited genes associated with the immune reaction. In addition, multiple factors have been identified as being involved in the control of alternative splicing at the *cis*, *trans*, or co-transcriptional level, and the aberrant splicing of RNA leads to the abnormal modulation of immune activity in infections, immune diseases, and tumors. In this review, we summarize the recent discoveries on the generation of immune-associated alternative splice variants, clinical disorders, and possible regulatory mechanisms. We also discuss the immune responses to the neoantigens produced by alternative splicing, and finally, we issue some alternative splicing and immunity correlated questions based on our knowledge.

## 1. Introduction

The coding sequence of eukaryotic genes is composed of exons and is formed by the removal of introns, as a result of pre-mRNA splicing. Alternative splicing enables the production of various RNA isoforms from a single gene by skipping or retaining some alternative exons. In humans, high-throughput genome-wide analyses have shown that about 95% of multi-exonic genes undergo alternative splicing, and about 37% of them generate multiple protein isoforms [1,2,3]. The diverse products resulting from alternative splicing are involved in the biological evolution of humans, especially for the function of the human brain [4,5,6], and play a mostly complementary role for the genome to define individual phenotypes in cell differentiation, aging, and sexual dimorphism [7,8,9].

Alternative RNA splicing and the surrounding environment of the organism have a mutual effect on each other. For example, the virus protein, NSP16, of SARS-CoV-2 has been found to bind to host splicing factors, U1 and U2, and globally suppress the mRNA splicing of infected cells [10]. During the global COVID-19 pandemic of SARS-CoV-2, scientists also tried to minimize the damage caused by the virus through modulating the alternative splicing of the ACE2 primary RNA, encoding the cellular receptor for the virus, to exclude critical domains required for virus entry, while keeping the vital physiological functional domains of ACE2 in human cells [11]. By evoking the genome to produce novel splice variants in response to stress, alternative splicing can also promote the ability of organisms to adapt to new challenges under the limitation of a constant genome size.

Human immunity is a complex process carried out by the innate and adaptive immune systems responding to surroundings, hormones, and psychosis [12,13,14]. About 60% of genes in lymphocytes express different splicing isoforms, and the alternative splicing of the pre-mRNA has been found to be deeply involved in the differentiation of immune cells and the regulation of immune response [15,16,17]. How alternative splicing regulates immunity is an interesting topic, both in biological and in clinical research. This review focuses on the research progress of alternative splicing in the human immune response and immune-related diseases, as well as the possible regulatory mechanisms of alternative splicing in recent years.

## 2. Influence of Alternative Splicing on Innate Immunity

Dendritic cells are considered the initiators of innate immunity and professional antigen presenting cells for the activation of adaptive immunity. Splicing-sensitive arrays indicate widespread alternative splicing events in human dendritic cells induced by bacterial challenge. These alternatively spliced genes are involved in dendritic cell development, endocytosis, and antigen presentation [18]. Macrophages are derived from blood monocytes and are involved in detection, phagocytosis, and cytokine secretion. Thousands of alternative splicing alterations have been detected when inducing the cells differentiation and activation in vitro [19]. The killer-cell immunoglobulin-like receptors (KIRs) expressed on natural killer (NK) cells mediate the interaction of major histocompatibility complex (MHC) class I molecules on most healthy cells. KIR isoforms produced by alternative splicing contribute to the complexity of the KIR gene system of NK cells to modulate NK cell killing function in the innate immune response [20,21].

Differentiated innate immune cells identify evolutionarily conserved components expressed by pathogens through pattern recognition receptors. After recognition of a pathogen, MyD88 protein acts as an adapter to connect the signals received from outside and the signals transferred to the inside of the cell. Under the control of splicing complexes, SF3A and SF3B, the *MyD88* gene undergoes alternative splicing to produce a long isoform, MyD88L, and short isoform, MyD88S, which play a role in activating and inhibiting the innate immunity, respectively, of innate immune cells by regulating Toll-like receptor signals [22,23]. Upon activation, intracellular signaling cascades are triggered to promote the expression of proinflammatory molecules, interferons (IFN), interleukins (IL), and chemokines involved in the clearance of antigens.

During innate immunity against microbes, specifically viruses, IFN produced by host cells had attracted much attention for the regulation of IFN production and signaling. The network of IFN-stimulated genes (ISGs) was driven by IFN secretion to establish an antiviral state. ISGs can also be antagonized by viruses for successful infection, while alternative splicing of ISGs in host cells can alter the localization and activity of ISG proteins to play a vital role in the control of host innate immunity to viral infection [24]. Zinc finger RNA-binding protein (ZFR) is a potent regulator of alternative splicing and can suppress the IFN response through regulating the splicing and activity of ISGs. Moreover, ZFR itself is also expressed in various isoforms to carry out different functions. Monocytes express truncated ZFR isoforms, and macrophages mainly express full-length ZFR to guard the IFN responses [25]. *SAT1* is also an antivirus associated gene, and SAT1 transcripts in virus-infected cells display higher inclusion of exon 4 to express an isoform of SAT1 that is generally degraded by nonsense mediated decay, thereby facilitating the infection by virus in cells [26]. There are also many other genes with alternative splicing variants that have been predicted to potentially regulate innate immunity [27,28]. Though elucidating the details of the mechanism will require more studies, it is believed that alternative splicing is broadly involved in the innate immunity of host-pathogen interactions. On one hand, host cells produce splice variants to inhibit the invasion and replication of microbes. On the other hand, viral infection results in the production of aberrant isoforms of antiviral genes to facilitate the invasion and replication of viruses.

## 3. Contribution of Alternative Splicing to Humoral Immunity

Humoral immunity is mediated by antibodies, which are produced by B lymphocytes. Upon maturation, B lymphocytes produce IgM as the first type of immunoglobulin, followed by immunoglobulin IgG, IgE, and IgA, as a result of irreversible rearrangement of the immunoglobulin gene after activation. In contrast, the IgM-to-IgD transition is produced through alternative splicing of a long primary mRNA transcript from the Ig heavy chain gene, *IGH* [29,30]. The nuclear RNA-binding protein, HuR, is well known for its function in mRNA splicing and nuclear export. HuR deficiency in B cells affects the proliferation and differentiation of B lymphocytes by controlling the splicing of hundreds of genes, including the IgM-to-IgD antibody class switch [31].

## 4. Control of T Cell Development and Activation Mediated by Splicing Isoforms

**Figure 1:**
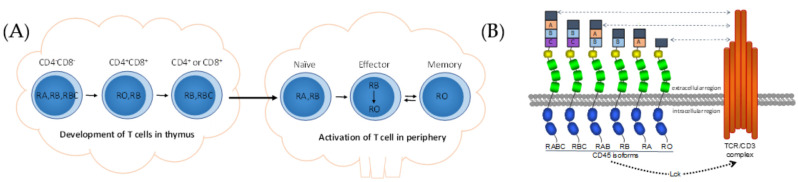
Expression and function of CD45 isoforms on T cells. (**A**) Expression of CD45 isoforms on the surface of T lymphocytes in thymus and peripheral lymphoid tissues. (**B**) Relationship of CD45 isoforms to the T cell receptor (TCR).

T cells develop from hematopoietic stem cells in the thymus and express the lineage-specific biomarker of T cell receptors on the cell membrane. A few notable alternatively spliced molecules have been clearly identified as being involved in the development and functional regulation of T cells. The spliced isoforms of transmembrane protein tyrosine phosphatase, CD45, are the first and most studied molecules in T cells [32,33]. The *CD45* gene can express isoforms CD45RA, CD45RB, CD45RC, and CD45RO through alternative exons (exons 4, 5, and 6) within the extracellular domain. The different CD45 isoforms are expressed at different developmental stages of T lymphocytes in the thymus during the negative and positive selection of the cells [34,35], and also correspond to different functional statuses of T cells after they face the antigens in peripheral lymphoid tissues [36,37] (Figure 1A). Though different CD45 isoforms are found in different subsets of the T cells, it is hard to say if the isoforms result in the differentiation of T cells. In recent years, light has been thrown on the interaction and function of CD45 with the T cell receptor (TCR). Researchers found that CD45 was involved in the formation of the immune synapse for initiating the activation of the TCR through cytoplasmic domains of CD45 to dephosphorylate the tyrosine kinase, Lck [38,39]. In the later stage of the signal transduction of T cell activation, TCRs are excluded from CD45 for their optimal activation. The differentially spliced CD45 isoforms have different sizes. The larger variant, RABC (approximately 40 nm for ectodomains), is excluded more rapidly and efficiently than the smaller isoform, RO (approximately 25 nm for ectodomains), from the TCR [40,41,42] (Figure 1B). These studies indicate that the structure of the extracellular domains of CD45, encoded by alternative splicing, controls the TCR signaling during the activation of T cells.

CD6 is another transmembrane glycoprotein expressed by medullary thymocytes and all mature T cells. Human CD6 mRNA consists of 13 exons, among which 7 exons at the 5′ end encode the extracellular region and transmembrane domain, and the 6 exons at the 3′ end encode the cytoplasmic domain [43,44,45]. The alternative splice sites of CD6 are located in nucleotide sequences for both the intracellular and extracellular domains. The isoform CD6Δ3 is produced from exon 5 skipping and consequently lacks the extracellular third scavenger receptor cysteine-rich domain, which can bind to CD166 on antigen presenting cells. Thus, the splicing out of exon 5 results the CD6Δd3, which is unable to localize at the T cell-antigen presenting cell interface, during the process of antigen presentation. Immunofluorescence assays indicate that after the activation of T cells, a portion of full-length CD6 was substituted by the CD6Δd3 isoform [46,47]. This mechanism controlling the expression of the CD166 binding domain through alternative splicing may help regulate signaling, delivered by CD6 during T cell activation. Based on the cytoplasmic regions, the isoforms of CD6 were identified and named as CD6a, CD6b, CD6c, CD6d, CD6e, and CD6f, and each isoform is formed by alternative splicing of the cytoplasmic encoding exons [48,49]. As an early consequence of T cell activation through the TCR complex, the cytoplasmic domains of CD6 undergo hyperphosphorylation, then interact with multiple kinases of different families within the cell [50,51]. The existence of these isoforms enables CD6 to flexibly regulate signal transduction and T cell function.

Besides the modulation of multiple CD molecules, cytotoxic T cell immune response is also correlated with a number of cytokines. IL-15 isoforms can impair IFN-γ production upon TCR engagement in mice [52]. Skipping of exon 5 in IL-7 leads to increased STAT5 signaling via IL-7R, to promote T cell survival [53]. Moreover, the IL-7 isoforms have also been identified with the ability to down-regulate splicing regulators, leading to the inclusion of exon 6 of CD95 on memory CD4+ T cells [54]. Bose et al. recently found that the global splicing efficiency of T cells became optimal within 2 h but declined between 4 and 6 h after the stimulation of T cells with phorbol myristate acetate, leading to the altered expression of IL-2 [55]. These results suggest that in the immnue response, interleukins might regulate or serve as targets for alternative splicing in the modulation of T cell function.

## 5. Abnormally Spliced Isoforms in Immune-Associated Diseases

Aberrant expression of alternatively spliced variants has been found to be associated with almost all the immune-associated diseases. One of the situations is infection. For example, various spliced forms of IL-7 have been identified in HIV patients, granulomatous lesions of mycobacteria tuberculosis patients, and tumor cell lines [53,54]. Another situation is the breaking of immune tolerance by aberrant splicing. Full-length IL-33 is a nuclear protein and may function as an alarm signal, released during cell death. While the splicing out of exons 3 and 4 confers cytoplasmic localization and facilitates extracellular secretion, that spliced isoform, not full-length IL-33, is strongly associated with airway type 2 inflammation in allergic airway diseases [56,57]. Autoimmune diseases also result from the imbalance of immune homeostasis. The alternatively spliced CD45RO isoform has been found to be increased in systemic lupus erythematosus (SLE), and CD45 isoforms have been suggested as biomarkers of SLE and are capable of distinguishing the functional status of T lymphocytes [58]. Several meta-analyses of genome-wide association screens on multiple sclerosis (MS) patients, and healthy controls, demonstrated significant association of single nucleotide polymorphisms correlated with CD6 alternative splicing and MS [59,60,61]. For the expression of CD6 isoforms in different immune activities, CD6 has also been suggested to be a potential target for the therapy of MS [62,63]. CD21 is membrane glycoprotein binding to the complement C3d fragment, which provides the co-stimulatory signal for the survival and activation of B cells [64,65]. The mature B cells express the short CD21 (CD21S) lacking exon 10a, and follicular dendritic cells selectively express long isoforms of CD21 (CD21L) containing exon 10a [66]. In clinic, CD21S+ expression was proposed to be used as a prognostic factor for better survival of the patients of diffuse large B-cell lymphoma, and CD21L+ in rheumatoid synovia indicated high inflammation [67,68], while the regulatory mechanism for the splicing isoforms has not been reported.

Tumor pathogenesis is complex, and it is also correlated with the surveillance of the immune system. Alternative splicing of pre-mRNAs is associated with tumor cell signal transduction in regulation of mitosis and migration, or helping tumor cells escape from immune surveillance, which has been well summarized in previously published reviews [69,70,71,72]. Additional knowledge of cancer type-specific changes in transcript splicing provides valuable clues for physicians in prognosing, diagnosing, and implementing therapeutic strategies for cancer.

## 6. Regulatory Mechanisms of Alternative Splicing in the Immune Response

Studies in the recent several decades have demonstrated that multiple factors have been involved in the alternative splicing at *cis*, *trans*, or co-transcriptional levels.

**Figure 2:**
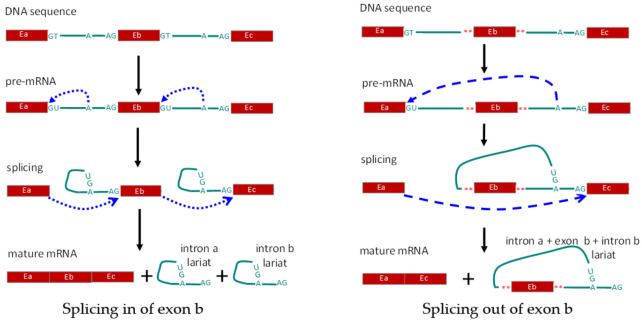
Schematic diagram showing regulation of alternative splicing in *cis*. Left panel, splicing in of exon b (Eb). Right panel, alternative splicing leading to removal of exon b. E: exon; A: branch site for RNA splicing; **: mutated nucleotides.

**Figure 3:**
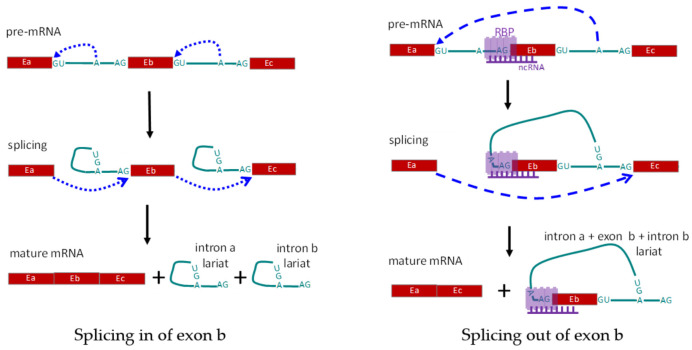
Schematic diagram showing regulation of alternative splicing in *trans*-act. Left panel shows the normal splicing to include all three exons. Right panel shows exclusion of exon Eb, due to alternative splicing mediated by RNA-binding protein(s), RBP(s), and non-coding RNA. E: exon; A: branch site for RNA splicing; RBP: RNA-binding protein(s); ncRNA: non-coding RNA complementary to the pre-mRNA.

**Figure 4:**
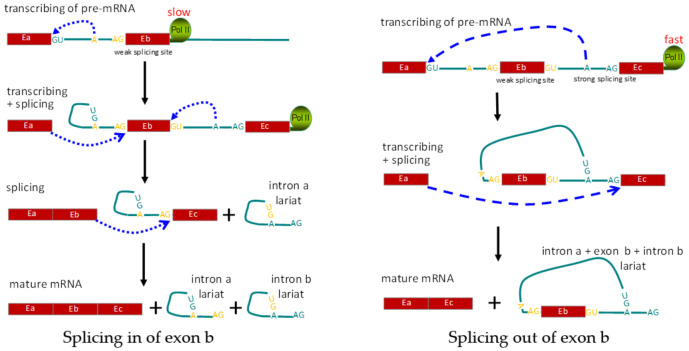
Schematic diagram showing regulation of alternative splicing at the co-transcriptional level. Left panel shows the normal splicing to include all three exons. Right panel shows exclusion of exon Eb, due to alternative splicing mediated by RNA Pol II speed. E: exon; A: branching site for RNA splicing; Pol II: RNA polymerase II.

(1) *Cis* regulation: *cis*-elements for splicing refer to the sequences in the pre-mRNA that correlate with the modulation of splicing, which include the GU sequence at the 5′ end as the splice donor site, the AG sequence at the 3′ end as the splice acceptor site, and the adenine branch point for definition of the exon and intron by the spliceosome. Also included are exonic splicing enhancers, exonic splicing silencers, intronic splicing enhancers, and intronic splicing silencers [4,73,74]. Alternative exons are generally associated with weak splice site signals [75]. Mutations in DNA might disrupt existing splice sites or create new splice sites and result in alternative splicing of pre-mRNA (Figure 2). C77G variation in exon 4 of the human *CD45* gene was identified as being correlated with the altered expression of CD45 isoforms and modulated function of T cells [76,77,78]. Besides DNA mutation, RNA editing can directly influence the splicing. N6-methyladenosine (m6A) represents one of the most abundant and dynamic RNA modifications in eukaryotes and controls the RNA structure and interactions with RNA-binding proteins [79]. The m6A reader and moderator, ALKBH5, can specifically remove m6A from target mRNAs. ALKBH5-mediated m6A erasure was found essential for correct RNA splicing in spermatocytes and spermatids, and failure to do so led to the production of aberrant splice variants that were rapidly degraded [80]. The infection by viruses, including dengue, Zika, West Nile, and hepatitis C viruses, alters m6A modification of RNA transcripts, with different viruses having different effects on host RNAs through promoting translation or alternative splicing to regulate infection [81].

(2) *Trans* regulation: *trans*-acting factors, formerly referred to as splicing factors, are RNA binding factors, including RNA binding proteins (RBPs) and noncoding RNA, that are involved in the formation of the spliceosome and directly take part in the splicing process (Figure 3). The proteins directly involved in the splicing are also called splicing factors, and the binding profile and functions of splicing factors have been broadly studied [75,82]. Ribonucleoprotein (hnRNP) family member, hnRNP L, and its cell-type specific paralog, hnRNP L-like (hnRNP LL), are some of the *trans*-acting factors first identified in repressing the splicing of the variable exons in the *CD45* gene. Knockdown of the expression level of hnRNP proteins, in vitro or in vivo, leads to altered CD45 pre-mRNA splicing and increased expression of CD45RA in human lymphoid cell lines. Exon array analysis showed that hnRNP LL acts as a global regulator of alternative splicing in activated T cells [83,84,85]. Besides the hnRNP family, the serine/Arginine family is another group of splicing factors, which comprises a number of structurally conserved proteins with arginine and serine residues in the domains. The member Serine/Arginine splicing factor 1 (SRSF1) has been found abundantly expressed in most tissues, acting as a master controller of T cell activity, and being involved the immune regulation of SLE [86,87,88]. During the alternative splicing of exon 5 in CD6, SRSF1 binds to the regulatory sequence in intron 4 and promotes the inclusion of exon 5. When the T cell has been activated, the binding level of SRSF1 to the CD6 pre-mRNA decrease, and exon 5 is prone to be skipped [89]. Tang et al. found that SRSF1 could promote the exclusion of exon 13 in CD46, which is a membrane protein to protect host cells from complements induced injury [90]. RNA binding proteins, PTBP1 and PTBP3, are expressed on B cells and belong to the PTB protein family, which specially bind to the pyrimidine-rich sequence of target RNAs and control the posttranscriptional splicing. PTBP1 was found having a regulatory effect on the splicing of CD46, while promoting the inclusion of exon 13 [90]. Genome-wide analysis indicates that the regulatory effect of PTB protein on exon splicing is up the binding position. When the proteins crosslink to the 3′UTR of the transcript, they can modulate RNA variants’ degradation by nonsense-mediated mRNA decay, thus suppressing the expression of target genes [91,92]. Experiments with an animal model indicate that PTBP proteins are essential for the development and antibody production of B cells [93,94,95]. In addition, the splicing factor, RBM10, is also found to be involved in the alternative splicing following viral infection, tumor progression, and inflammation development [26,96,97,98]. Recently, multiple RNA sites for RBPs have been identified in the ENCODE phase III project, after the typical ENCODE phase I and II projects [99]. The binding sites influence the binding and function of splicing factors, and ultimately control the post-transcriptional processes including RNA splicing.

(3) Co-transcriptional regulation: co-transcriptional regulation differs from *cis* and *trans* regulation, since there are no direct alterations of pre-RNA sequences and RNA-binding proteins. During co-transcriptional regulation, the transcriptional process regulates post-transcriptional splicing, mainly through controlling the speed of RNA polymerase II [100,101]. DNA methylation in promoters is well established with repressed activity of transcriptional initiation, while DNA methylation in gene bodies serves as the foundation to block the binding of CTCF, resulting in faster movement of RNA polymerase II and the limited time for recognition of the weak splice alternative exon 5 in the *CD45* gene, resulting in increased skipping of alternative exons [102]. A series of histone modifications on the alternative exon’s region was also correlated with the polymerase II elongation rate and alternative splicing of the CD6 and CD44 genes [89,103]. No matter whether it’s due to DNA methylation or histone modification, a similar mechanism is involved whereby the polymerase II elongation rate is modified to affect alternative splicing of exons at the co-transcriptional level (Figure 4).

Among the splicing regulators, non-coding RNAs, including linear long non-coding RNA (lncRNA), linear small non-coding RNA (sncRNA), and circular RNA (circRNA), are also considered to play an important role in splice regulation at the *trans* or co-transcriptional level. LncRNAs are usually generated endogenously from the genome and lack significant protein coding capacity. MALAT1 (the metastasis-associated lung adenocarcinoma transcript 1, also known as NEAT2) is a highly conserved lncRNA and predominantly localizes within the nucleus, where it regulates the splicing of numerous genes by interaction with splicing factors or other miRNAs [104,105]. Besides being associated with multiple tumors, MALAT1 was also found with altered expression in the infectious disease [106,107], immune associated atherosclerosis [108], and asthma [109] in recent years. Among the endogenous lncRNAs, natural antisense transcripts (NATs) are transcribed from DNA strands at the same locus as the sense gene, but in the opposite direction. Global transcriptome analysis of lymphoblastoid cell lines manifested that about 40% of human genes have NATs, and these NATs are affected by alternative splicing of exons in about 75% of the sense-antisense annotations [110,111]. The NAT of CD45 was proposed with the potential to regulate the alternative splicing of CD45 at a co-transcriptional level through recruiting factors to modify the DNA methylation of sense genes [112,113].

SncRNAs are generated from lncRNAs or directly transcribed from protein-coding or nonprotein-coding transcriptional units. In the nucleus, a special group of sncRNAs directly takes part in the formation of the spliceosome together with splicing factors and is involved in the catalysis of the splicing reaction [114,115]. U1 small nuclear RNA was identified as being involved in defining exons 7 and 8 of CD46 [90]. Through dissection of the high-throughput data of SARS-CoV-2 viral bound human proteins and computed analysis of the alternative splicing events, Srivastava et al. revealed that SARS-CoV-2 interacted with 51 human RBPs and 22 miRNAs of host cells, of which the majority of them were highly expressed in gonadal tissues and immune cells, including T cells, NK cells, and monocytes [116,117]. The results indicate that one microbe can interact both with RBPs and noncoding RNAs to alter post-transcriptional regulation in specialized immune cells.

CircRNAs are a newly recognized class of closed noncoding RNAs, in which the 5′ and 3′ termini are covalently linked by back-splicing of exons from pre-mRNA. During back-splicing, the internal exons of pre-mRNA are excised to form a circRNA, and the remaining pre-mRNA are spliced in an alternative pattern to form linear RNA, which indicates the back-splicing of circRNA competes with the canonical splicing of their linear equivalents [118]. Moreover, circRNAs are relatively stable in the cells to serve as miRNA sponges and contribute to modulating gene expression [119,120]. The circPan3 generated from the linear primary transcript of the Pan3 gene increases the expression of IL-13 in small intestinal immune cells through binding to and stabilizing the mRNA of IL-13Rα1 [121]. Based on the interaction with miRNAs and splicing competition for linear RNA production, the expression of circRNAs was correlated with the pathogenesis of multiple tumors, T cell ageing, virus infection, and autoimmune diseases [122,123,124].

The summary of the splicing regulators (Table 1) indicates that the alternative splicing of a single gene, for example CD45 and CD46, could be regulated in multiple ways in different situations. Both proteins and noncoding RNAs can regulate the splicing at the *trans*-act or co-transcriptional level. The proteins generally affect alternative splicing on a broad, or even globally, genomic scale. While alternative splicing is regulated by exogenously synthesized oligo RNAs or endogenous natural antisense transcripts, greater target site specificity occurs due to sequence guided complementation. The first application of antisense RNA in clinic was for the treatment of patients with spinal muscular atrophy [125,126,127]. This should also be a promising therapeutic strategy for delivering splice-switching oligomers to lymphoid cells for splicing-associated immune diseases by correcting mis-splicing or altering the balance of different splice isoforms.

## 7. Neoantigens Produced by Alternative Splicing

Neoantigens are novel peptides with epitopes or antigenic determinants to the immune system, which are generated in specific situations, for example during the pathogenesis and progression of tumors. All the DNA mutation, RNA editing, and post-transcriptional splicing have the possibility to produce neoantigens [128,129], and several types of software have been reported for the prediction of neoantigens, based on the information from RNA-seq data [130,131,132,133]. In normal cells, not all of these aberrant RNA variants have the possibility to produce protein isoforms. It was proposed that about 30% of alternatively spliced transcripts in human cells contain premature termination codons that will result in the degradation of the RNA through nonsense-mediated decay [134]. Moreover, great numbers of these RNA variants are noncoding RNA. However, the nonsense-mediated decay regulation mechanism is also frequently disrupted in cancer cells, which allowing the aberrant transcripts to escape degradation [135,136]. Therefore, accumulations of aberrant transcripts are frequently observed in cancer cells.

The neoantigens identified from tumor patients of multiple myeloma have been verified in vitro by experiments with potential to activate neoantigen specific T cells [137]. Tumor cells in vivo have a series of ways to escape the surveillance of the immune system, including low expression of MHC for the poor antigen presentation, excluding of effector cells from the invasion of tumors, and being replaced with immune suppressor cells [138]. This condition promotes the development of specific tumor vaccines, and tumor targeted therapy strategies, such as anti-CTLA-4 and anti-PD-1, have been applied in clinic in recent years as precision medicine and achieved many successes [139,140].

## 8. Prospect

We present a short review concerning alternative splicing in immune activity. In addition, we also pose some questions associated with splicing and list them here for researchers having the same interests for further discussion and exploration.

(1) Tumor cells have strategies to escape immune surveillance of neoantigens generated from the spliced variants, while for the normal somatic cells, there are also novel protein variants produced to deal with altered exogeneous surroundings or endogenous modulation. How does the immune system recognize and deal with these novel protein isoforms in the life span?
(2) What are the physiological functions of the spliced isoforms? Most human genes have alternatively spliced RNA isoforms. Presently, only a small portion of them correlate with clinical disorders, such as cancer, heart disease, and immune disease, and have attracted the attention of scientific researchers, while the functions of most of them has not been identified. It is considered that spliced isoforms might play an important role through providing diversity transcripts in the long-term evolution of immune responses [141]. If without function, will these alternative exons be removed as junk sequences in the future evolution of humans?
(3) If alternative splicing is correlated with clinical diseases, the next step will be how to target the abnormal alternative splicing to provide treatment. For refractory autoimmune diseases, besides the application of chemical medicines, whether the production of the alternative splicing be prevented or be targeted for degradation should be a question worthy of consideration and exploration.

In summary, immune activity is an extremely complex response to exogenous surroundings and endogenous regulation involving multiple factors. Alternative splicing, by producing RNA or protein isoforms, plays a crucial role in the immune response from the physiological development of immune cells and regulation of immune activity, to clinical immune-associated disorders. There are also some questions, as mentioned above, about the relationship between immunity and alternative splicing, which is waiting for answers from the scientific progress of related fields. Nevertheless, antisense oligonucleotides have a high potential for modulating alternative splicing in future clinical trials, since they have the potential to target specific DNA or RNA sequences.

## Figures and Tables

**Table 1:** The representative splicing regulators associated with immunity.

Regulator	Acting Way	Target Gene	Associated Function	Ref.
DNA mutation—C77G	*Cis*	*CD45*	Mutation in DNA sequence of *CD45* exon 4 alters the splicing of the exon.	[78]
RNA editing—M6A	*Cis*	*CIRBP*	Viral infection modulates alternative splicing of *CIRBP* through RNA modification.	[81]
RBP—hnRNP LL	*Trans*	*CD45*	HnRNP LL protein binds to *CD45* transcript to control the expression of CD45RA and RO.	[84]
RBP—SRSF1	*Trans*	*CD46*	SRSF1 protein promotes the exclusion of exon 13 in *CD46*.	[90]
RBP—PTBP1	*Trans*	*CD46*	PTBP1 protein promotes the inclusion of exon 13 in *CD46*.	[90]
RBP—RBM10	*Trans*	*SAT1*	RBM10 is responsible for *SAT1* exon 4 skipping for limiting viral replication.	[26]
lncRNA—MALAT1	*Trans*	*SAT1*	MALAT1 regulates the splicing of multiple genes including *SAT1* by interaction with serine/arginine splicing factors.	[104]
sncRNA—U1 RNA	*Trans*	*CD46*	U1 small nuclear RNA defines exons 7 and 8 of *CD46*.	[90]
circRNA—circPan3	*Trans*	*Pan*	circPan3, originated from the back-splicing of *Pan3* transcript, increases the expression of IL-13.	[121]
DNA modification	co-transcriptional	*CD45*	DNA binding protein, CTCF, linking DNA methylation to modulate the splicing of *CD45* through controlling the RNA polymerase II elongation rate.	[102]
Histone modification	co-transcriptional	*CD44*	Histone modification modulates the alternative splicing of *CD44* through controlling the RNA polymerase II elongation rate.	[103]

## Data Availability

Not applicable.

## References

[1] Mele M., Ferreira P.G., Reverter F., DeLuca D.S., Monlong J., Sammeth M., Young T.R., Goldmann J.M., Pervouchine D.D., Sullivan T.J. (2015). Human genomics. The human transcriptome across tissues and individuals. Science.

[2] Wang E.T., Sandberg R., Luo S., Khrebtukova I., Zhang L., Mayr C., Kingsmore S.F., Schroth G.P., Burge C.B. (2008). Alternative isoform regulation in human tissue transcriptomes. Nature.

[3] Kim M.S., Pinto S.M., Getnet D., Nirujogi R.S., Manda S.S., Chaerkady R., Madugundu A.K., Kelkar D.S., Isserlin R., Jain S. (2014). A draft map of the human proteome. Nature.

[4] Keren H., Lev-Maor G., Ast G. (2010). Alternative splicing and evolution: Diversification, exon definition and function. Nat. Rev. Genet..

[5] Xiong J., Jiang X., Ditsiou A., Gao Y., Sun J., Lowenstein E.D., Huang S., Khaitovich P., Xi J. (2018). Predominant patterns of splicing evolution on human, chimpanzee and macaque evolutionary lineages. Hum. Mol. Genet..

[6] Berto S., Mendizabal I., Usui N., Toriumi K., Chatterjee P., Douglas C., Tamminga C.A., Preuss T.M., Yi S.V., Konopka G. (2019). Accelerated evolution of oligodendrocytes in the human brain. Proc. Natl. Acad. Sci. USA.

[7] Baralle F.E., Giudice J. (2017). Alternative splicing as a regulator of development and tissue identity. Nat. Rev. Mol. Cell Biol..

[8] Bhadra M., Howell P., Dutta S., Heintz C., Mair W.B. (2020). Alternative splicing in aging and longevity. Hum. Genet..

[9] Rogers T.F., Palmer D.H., Wright A.E. (2021). Sex-Specific selection drives the evolution of alternative splicing in birds. Mol. Biol. Evol..

[10] Banerjee A.K., Blanco M.R., Bruce E.A., Honson D.D., Chen L.M., Chow A., Bhat P., Ollikainen N., Quinodoz S.A., Loney C. (2020). SARS-CoV-2 disrupts splicing, translation, and protein trafficking to suppress host defenses. Cell.

[11] Rehman S.U., Tabish M. (2020). Alternative splicing of ACE2 possibly generates variants that may limit the entry of SARS-CoV-2: A potential therapeutic approach using SSOs. Clin. Sci..

[12] Kim C.H. (2018). Immune regulation by microbiome metabolites. Immunology.

[13] Bansal A., Henao-Mejia J., Simmons R.A. (2018). Immune system: An emerging player in mediating effects of endocrine disruptors on metabolic health. Endocrinology.

[14] Muscatell K.A. (2020). Social psychoneuroimmunology: Understanding bidirectional links between social experiences and the immune system. Brain Behav. Immun..

[15] Ergun A., Doran G., Costello J.C., Paik H.H., Collins J.J., Mathis D., Benoist C., Blair D.A., Dustin M.L., Shinton S.A. (2013). Differential splicing across immune system lineages. Proc. Natl. Acad. Sci. USA.

[16] Martinez N.M., Lynch K.W. (2013). Control of alternative splicing in immune responses: Many regulators, many predictions, much still to learn. Immunol. Rev..

[17] Yabas M., Elliott H., Hoyne G.F. (2015). The role of alternative splicing in the control of immune homeostasis and cellular differentiation. Int. J. Mol. Sci..

[18] Rodrigues R., Grosso A.R., Moita L. (2013). Genome-Wide analysis of alternative splicing during dendritic cell response to a bacterial challenge. PLoS ONE.

[19] Liu H., Lorenzini P.A., Zhang F., Xu S., Wong M.S.M., Zheng J., Roca X. (2018). Alternative splicing analysis in human monocytes and macrophages reveals MBNL1 as major regulator. Nucleic Acids Res..

[20] Korbel D.S., Norman P.J., Newman K.C., Horowitz A., Gendzekhadze K., Parham P., Riley E.M. (2009). Killer Ig-like receptor (KIR) genotype predicts the capacity of human KIR-positive CD56dim NK cells to respond to pathogen-associated signals. J. Immunol..

[21] Bruijnesteijn J., van der Wiel M.K.H., de Groot N., Otting N., de Vos-Rouweler A.J.M., Lardy N.M., de Groot N.G., Bontrop R.E. (2018). Extensive alternative splicing of KIR transcripts. Front. Immunol..

[22] Pizzolla A., Smith J.M., Brooks A.G., Reading P.C. (2017). Pattern recognition receptor immunomodulation of innate immunity as a strategy to limit the impact of influenza virus. J. Leukoc. Biol..

[23] De Arras L., Alper S. (2013). Limiting of the innate immune response by SF3A-dependent control of MyD88 alternative mRNA splicing. PLoS Genet..

[24] Savan R. (2018). Alternative splicing in innate antiviral immunity. J. Interferon Cytokine Res..

[25] Haque N., Ouda R., Chen C., Ozato K., Hogg J.R. (2018). ZFR coordinates crosstalk between RNA decay and transcription in innate immunity. Nat. Commun..

[26] Pozzi B., Bragado L., Mammi P., Torti M.F., Gaioli N., Gebhard L.G., Solá M.E.G., Vaz-Drago R., Iglesias N.G., García C.C. (2020). Dengue virus targets RBM10 deregulating host cell splicing and innate immune response. Nucleic Acids Res..

[27] Ashraf U., Benoit-Pilven C., Lacroix V., Navratil V., Naffakh N. (2019). Advances in analyzing virus-induced alterations of host cell splicing. Trends Microbiol..

[28] Frankiw L., Mann M., Li G., Joglekar A., Baltimore D. (2020). Alternative splicing coupled with transcript degradation modulates OAS1g antiviral activity. RNA.

[29] Maki R., Roeder W., Traunecker A., Sidman C., Wabl M., Raschke W., Tonegawa S. (1981). The role of DNA rearrangement and alternative RNA processing in the expression of immunoglobulin delta genes. Cell.

[30] Zhang K., Saxon A., Max E.E. (1992). Two unusual forms of human immunoglobulin E encoded by alternative RNA splicing of epsilon heavy chain membrane exons. J. Exp. Med..

[31] Diaz-Munoz M.D., Bell S.E., Fairfax K., Monzon-Casanova E., Cunningham A.F., Gonzalez-Porta M., Andrews S.R., Bunik V.I., Zarnack K., Curk T. (2015). The RNA-binding protein HuR is essential for the B cell antibody response. Nat. Immunol..

[32] Pilarski L.M., Gillitzer R., Zola H., Shortman K., Scollay R. (1989). Definition of the thymic generative lineage by selective expression of high molecular weight isoforms of CD45 (T200). Eur. J. Immunol..

[33] Chui D., Ong C.J., Johnson P., Teh H.S., Marth J.D. (1994). Specific CD45 isoforms differentially regulate T cell receptor signaling. EMBO J..

[34] Fukuhara K., Okumura M., Shiono H., Inoue M., Kadota Y., Miyoshi S., Matsuda H. (2002). A study on CD45 isoform expression during T-cell development and selection events in the human thymus. Hum. Immunol..

[35] Hermiston M.L., Xu Z., Weiss A. (2003). CD45: A critical regulator of signaling thresholds in immune cells. Annu. Rev. Immunol..

[36] Andersen M.H., Schrama D., Thor Straten P., Becker J.C. (2006). Cytotoxic T cells. J. Investig. Dermatol..

[37] Zikherman J., Weiss A. (2008). Alternative splicing of CD45: The tip of the iceberg. Immunity.

[38] Cordoba S.P., Choudhuri K., Zhang H., Bridge M., Basat A.B., Dustin M.L., Van Der Merwe P.A. (2013). The large ectodomains of CD45 and CD148 regulate their segregation from and inhibition of ligated T-cell receptor. Blood.

[39] Chang V.T., Fernandes R.A., Ganzinger K.A., Lee S.F., Siebold C., McColl J., Jönsson P., Palayret M., Harlos K., Coles C.H. (2016). Initiation of T cell signaling by CD45 segregation at ‘close contacts’. Nat. Immunol..

[40] Carbone C.B., Kern N., Fernandes R.A., Hui E., Su X., Garcia K.C., Vale R.D. (2017). In vitro reconstitution of T cell receptor-mediated segregation of the CD45 phosphatase. Proc. Natl. Acad. Sci. USA.

[41] Razvag Y., Neve-Oz Y., Sajman J., Reches M., Sherman E. (2018). Nanoscale kinetic segregation of TCR and CD45 in engaged microvilli facilitates early T cell activation. Nat. Commun..

[42] Courtney A.H., Shvets A.A., Lu W., Griffante G., Mollenauer M., Horkova V., Lo W.L., Yu S., Stepanek O., Chakraborty A.K. (2019). CD45 functions as a signaling gatekeeper in T cells. Sci. Signal..

[43] Orta-Mascaro M., Consuegra-Fernandez M., Carreras E., Roncagalli R., Carreras-Sureda A., Alvarez P., Girard L., Simões I., Martínez-Florensa M., Aranda F. (2016). CD6 modulates thymocyte selection and peripheral T cell homeostasis. J. Exp. Med..

[44] Santos R.F., Oliveira L., Carmo A.M. (2016). Tuning T cell activation: The function of CD6 at the immunological synapse and in T cell responses. Curr. Drug Targets.

[45] Goncalves C.M., Henriques S.N., Santos R.F., Carmo A.M. (2018). CD6, a Rheostat-Type signalosome that Tunes T cell activation. Front. Immunol..

[46] Castro M.A., Oliveira M.I., Nunes R.J., Fabre S., Barbosa R., Peixoto A., Brown M.H., Parnes J.R., Bismuth G., Moreira A. (2007). Extracellular isoforms of CD6 generated by alternative splicing regulate targeting of CD6 to the immunological synapse. J. Immunol..

[47] Santos R.F., Oliveira L., Brown M.H., Carmo A.M. (2019). Domain-specific CD6 monoclonal antibodies identify CD6 isoforms generated by alternative-splicing. Immunology.

[48] Robinson W.H., Neuman de Vegvar H.E., Prohaska S.S., Rhee J.W., Parnes J.R. (1995). Human CD6 possesses a large, alternatively spliced cytoplasmic domain. Eur. J. Immunol..

[49] Kureel A.K., Kumari S., Saini S., Satyaprakash Singh B., Rai A.K. (2019). Identification of a novel transcript variant of the human CD6 gene that lacks exon 9. Immunobiology.

[50] Bowen M.A., Whitney G.S., Neubauer M., Starling G.C., Palmer D., Zhang J., Nowak N.J., Shows T.B., Aruffo A. (1997). Structure and chromosomal location of the human CD6 gene: Detection of five human CD6 isoforms. J. Immunol..

[51] Castro M.A., Nunes R.J., Oliveira M.I., Tavares P.A., Simoes C., Parnes J.R., Moreira A., Carmo A.M. (2003). OX52 is the rat homologue of CD6: Evidence for an effector function in the regulation of CD5 phosphorylation. J. Leukoc. Biol..

[52] Nishimura H., Yajima T., Naiki Y., Tsunobuchi H., Umemura M., Itano K., Matsuguchi T., Suzuki M., Ohashi P.S., Yoshikai Y. (2000). Differential roles of interleukin 15 mRNA isoforms generated by alternative splicing in immune responses in vivo. J. Exp. Med..

[53] Vudattu N.K., Magalhaes I., Hoehn H., Pan D., Maeurer M.J. (2009). Expression analysis and functional activity of interleukin-7 splice variants. Genes Immun..

[54] Yin Y., Zhang S., Luo H., Zhang X., Geng G., Li J., Guo X., Cai W., Li L., Liu C. (2015). Interleukin 7 up-regulates CD95 protein on CD4+ T cells by affecting mRNA alternative splicing: Priming for a synergistic effect on HIV-1 reservoir maintenance. J. Biol. Chem..

[55] Bose D., Neumann A., Timmermann B., Meinke S., Heyd F. (2019). Differential Interleukin-2 transcription kinetics render mouse but not human T cells vulnerable to splicing inhibition early after activation. Mol. Cell. Biol..

[56] Drake L.Y., Kita H. (2017). IL-33: Biological properties, functions, and roles in airway disease. Immunol. Rev..

[57] Gordon E.D., Simpson L.J., Rios C.L., Ringel L., Lachowicz-Scroggins M.E., Peters M.C., Wesolowska-Andersen A., Gonzalez J.R., MacLeod H.J., Christian L.S. (2016). Alternative splicing of interleukin-33 and type 2 inflammation in asthma. Proc. Natl. Acad. Sci. USA.

[58] Silva-Neta H.L., Brelaz-de-Castro M.C.A., Chagas M.B.O., Mariz H.A., de Arruda R.G., de Vasconcelos V.F., Pereira M.C., Romano A., Pitta I.R., Marques C.D.L. (2018). CD4(+)CD45RA(-)FOXP3(low) Regulatory T Cells as potential biomarkers of disease activity in systemic lupus erythematosus brazilian patients. BioMed Res. Int..

[59] De Jager P.L., Jia X., Wang J., de Bakker P.I., Ottoboni L., Aggarwal N.T., Piccio L., Raychaudhuri S., Tran D., International MS Genetics Consortium (2009). Meta-Analysis of genome scans and replication identify CD6, IRF8 and TNFRSF1A as new multiple sclerosis susceptibility loci. Nat. Genet..

[60] Swaminathan B., Matesanz F., Cavanillas M.L., Alloza I., Otaegui D., Olascoaga J., Cenit M.C., Heras V.D.L., Barcina M.G., Arroyo R. (2010). Validation of the CD6 and TNFRSF1A loci as risk factors for multiple sclerosis in Spain. J. Neuroimmunol..

[61] International Multiple Sclerosis Genetics Consortium (2011). The genetic association of variants in CD6, TNFRSF1A and IRF8 to multiple sclerosis: A multicenter case-control study. PLoS ONE.

[62] Li Y., Singer N.G., Whitbred J., Bowen M.A., Fox D.A., Lin F. (2017). CD6 as a potential target for treating multiple sclerosis. Proc. Natl. Acad. Sci. USA.

[63] Consuegra-Fernandez M., Isamat M., Lozano F. (2017). Commentary: CD6 as a potential target for treating multiple sclerosis. Front. Immunol..

[64] Fischer M.B., Goerg S., Shen L., Prodeus A.P., Goodnow C.C., Kelsoe G., Carroll M.C. (1998). Dependence of germinal center B cells on expression of CD21/CD35 for survival. Science.

[65] Kozono Y., Abe R., Kozono H., Kelly R.G., Azuma T., Holers V.M. (1998). Cross-linking CD21/CD35 or CD19 increases both B7-1 and B7-2 expression on murine splenic B cells. J. Immunol..

[66] Liu Y.J., Xu J., de Bouteiller O., Parham C.L., Grouard G., Djossou O., De Saint-Vis B., Lebecque S., Banchereau J., Moore K.W. (1997). Follicular dendritic cells specifically express the long CR2/CD21 isoform. J. Exp. Med..

[67] Ogawa S., Yamaguchi M., Oka K., Taniguchi M., Ito M., Nishii K., Nakase K., Ohno T., Kita K., Kobayashi T. (2004). CD21S antigen expression in tumour cells of diffuse large B-cell lymphomas is an independent prognostic factor indicating better overall survival. Br. J. Haematol..

[68] McKelvey K.J., Millier M.J., Doyle T.C., Stamp L.K., Highton J., Hessian P.A. (2018). Co-expression of CD21L and IL17A defines a subset of rheumatoid synovia, characterised by large lymphoid aggregates and high inflammation. PLoS ONE.

[69] Marzese D.M., Manughian-Peter A.O., Orozco J.I.J., Hoon D.S.B. (2018). Alternative splicing and cancer metastasis: Prognostic and therapeutic applications. Clin. Exp. Metastasis.

[70] Frankiw L., Baltimore D., Li G. (2019). Alternative mRNA splicing in cancer immunotherapy. Nat. Rev. Immunol..

[71] Bonnal S.C., Lopez-Oreja I., Valcarcel J. (2020). Roles and mechanisms of alternative splicing in cancer—Implications for care. Nat. Rev. Clin. Oncol..

[72] Wang Y., Zhang H., Jiao B., Nie J., Li X., Wang W., Wang H. (2020). The roles of alternative splicing in tumor-immune cell interactions. Curr. Cancer Drug Targets.

[73] Mishra S.K., Thakran P. (2018). Intron specificity in pre-mRNA splicing. Curr. Genet..

[74] Mishra S.K., Muthye V., Kandoi G. (2020). Computational methods for predicting functions at the mRNA isoform level. Int. J. Mol. Sci..

[75] Fu X.D., Ares M. (2014). Context-dependent control of alternative splicing by RNA-binding proteins. Nat. Rev. Genet..

[76] Tchilian E.Z., Wallace D.L., Imami N., Liao H.X., Burton C., Gotch F., Martinson J., Haynes B.F., Beverley P.C.L. (2001). The exon A (C77G) mutation is a common cause of abnormal CD45 splicing in humans. J. Immunol..

[77] Dawes R., Petrova S., Liu Z., Wraith D., Beverley P.C., Tchilian E.Z. (2006). Combinations of CD45 isoforms are crucial for immune function and disease. J. Immunol..

[78] Windhagen A., Sonmez D., Hornig-Do H.T., Kalinowsky A., Schwinzer R. (2007). Altered CD45 isoform expression in C77G carriers influences cytokine responsiveness and adhesion properties of T cells. Clin. Exp. Immunol..

[79] Liu N., Dai Q., Zheng G., He C., Parisien M., Pan T. (2015). N(6)-methyladenosine-dependent RNA structural switches regulate RNA-protein interactions. Nature.

[80] Tang C., Klukovich R., Peng H., Wang Z., Yu T., Zhang Y., Zheng H., Klungland A., Yan W. (2018). ALKBH5-dependent m6A demethylation controls splicing and stability of long 3’-UTR mRNAs in male germ cells. Proc. Natl. Acad. Sci. USA.

[81] Gokhale N.S., McIntyre A.B.R., Mattocks M.D., Holley C.L., Lazear H.M., Mason C.E., Horner S.M. (2020). Altered m(6)A modification of specific cellular transcripts affects flaviviridae infection. Mol. Cell.

[82] Ule J., Blencowe B.J. (2019). Alternative splicing regulatory networks: Functions, mechanisms, and evolution. Mol. Cell.

[83] Rothrock C.R., House A.E., Lynch K.W. (2005). HnRNP L represses exon splicing via a regulated exonic splicing silencer. EMBO J..

[84] Oberdoerffer S., Moita L.F., Neems D., Freitas R.P., Hacohen N., Rao A. (2008). Regulation of CD45 alternative splicing by heterogeneous ribonucleoprotein, hnRNPLL. Science.

[85] Preussner M., Schreiner S., Hung L.H., Porstner M., Jack H.M., Benes V., Rätsch G., Bindereif A., Preussner M. (2012). HnRNP L and L-like cooperate in multiple-exon regulation of CD45 alternative splicing. Nucleic Acids Res..

[86] Katsuyama T., Li H., Comte D., Tsokos G.C., Moulton V.R. (2019). Splicing factor SRSF1 controls T cell hyperactivity and systemic autoimmunity. J. Clin. Investig..

[87] Katsuyama T., Martin-Delgado I.J., Krishfield S.M., Kyttaris V.C., Moulton V.R. (2020). Splicing factor SRSF1 controls T cell homeostasis and its decreased levels are linked to lymphopenia in systemic lupus erythematosus. Rheumatology.

[88] Paz S., Ritchie A., Mauer C., Caputi M. (2021). The RNA binding protein SRSF1 is a master switch of gene expression and regulation in the immune system. Cytokine Growth Factor Rev..

[89] da Gloria V.G., Martins de Araujo M., Mafalda Santos A., Leal R., de Almeida S.F., Carmo A.M., Moreira A. (2014). T cell activation regulates CD6 alternative splicing by transcription dynamics and SRSF1. J. Immunol..

[90] Tang S.J., Luo S., Ho J.X.J., Ly P.T., Goh E., Roca X. (2016). Characterization of the regulation of CD46 RNA alternative splicing. J. Biol. Chem..

[91] La Porta J., Matus-Nicodemos R., Valentin-Acevedo A., Covey L.R. (2016). The RNA-binding protein, Polypyrimidine Tract-Binding Protein 1 (PTBP1) is a key regulator of CD4 T cell activation. PLoS ONE.

[92] Hu J., Qian H., Xue Y., Fu X.D. (2018). PTB/nPTB: Master regulators of neuronal fate in mammals. Biophys. Rep..

[93] Monzon-Casanova E., Screen M., Diaz-Munoz M.D., Coulson R.M.R., Bell S.E., Lamers G., Solimena M., Smith C.W.J., Turner M. (2018). The RNA-binding protein PTBP1 is necessary for B cell selection in germinal centers. Nat. Immunol..

[94] Sasanuma H., Ozawa M., Yoshida N. (2019). RNA-binding protein Ptbp1 is essential for BCR-mediated antibody production. Int. Immunol..

[95] Monzon-Casanova E., Matheson L.S., Tabbada K., Zarnack K., Smith C.W., Turner M. (2020). Polypyrimidine tract-binding proteins are essential for B cell development. eLife.

[96] Wang Y., Gogol-Doring A., Hu H., Frohler S., Ma Y., Jens M., Maaskola J., Murakawa Y., Quedenau C., Landthaler M. (2013). Integrative analysis revealed the molecular mechanism underlying RBM10-mediated splicing regulation. EMBO Mol. Med..

[97] Inoue A., Yamamoto N., Kimura M., Nishio K., Yamane H., Nakajima K. (2014). RBM10 regulates alternative splicing. FEBS Lett..

[98] Hernandez J., Bechara E., Schlesinger D., Delgado J., Serrano L., Valcarcel J. (2016). Tumor suppressor properties of the splicing regulatory factor RBM10. RNA Biol..

[99] Van Nostrand E.L., Freese P., Pratt G.A., Wang X., Wei X., Xiao R., Blue S.M., Chen J.-Y., Cody N.A.L., Dominguez D. (2020). A large-scale binding and functional map of human RNA-binding proteins. Nature.

[100] Shukla S., Oberdoerffer S. (2012). Co-transcriptional regulation of alternative pre-mRNA splicing. Biochim. Biophys. Acta.

[101] Davis-Turak J., Johnson T.L., Hoffmann A. (2018). Mathematical modeling identifies potential gene structure determinants of co-transcriptional control of alternative pre-mRNA splicing. Nucleic Acids Res..

[102] Shukla S., Kavak E., Gregory M., Imashimizu M., Shutinoski B., Kashlev M., Oberdoerffer P., Sandberg R., Oberdoerffer S. (2011). CTCF-promoted RNA polymerase II pausing links DNA methylation to splicing. Nature.

[103] Jimeno-Gonzalez S., Payan-Bravo L., Munoz-Cabello A.M., Guijo M., Gutierrez G., Prado F., Reyes J.C. (2015). Defective histone supply causes changes in RNA polymerase II elongation rate and cotranscriptional pre-mRNA splicing. Proc. Natl. Acad. Sci. USA.

[104] Tripathi V., Ellis J.D., Shen Z., Song D.Y., Pan Q., Watt A.T., Freier S.M., Bennett C.F., Sharma A., Bubulya P.A. (2010). The nuclear-retained noncoding RNA MALAT1 regulates alternative splicing by modulating SR splicing factor phosphorylation. Mol. Cell.

[105] Zhang X., Hamblin M.H., Yin K.J. (2017). The long noncoding RNA Malat1: Its physiological and pathophysiological functions. RNA Biol..

[106] Hewitson J.P., West K.A., James K.R., Rani G.F., Dey N., Romano A., Brown N., Teichmann S.A., Kaye P.M., Lagos D. (2020). Malat1 suppresses immunity to infection through promoting expression of maf and IL-10 in Th cells. J. Immunol..

[107] Liu W., Wang Z., Liu L., Yang Z., Liu S., Ma Z., Liu Y., Ma Y., Zhang L., Zhang X. (2020). LncRNA Malat1 inhibition of TDP43 cleavage suppresses IRF3-initiated antiviral innate immunity. Proc. Natl. Acad. Sci. USA.

[108] Gast M., Rauch B.H., Nakagawa S., Haghikia A., Jasina A., Haas J., Nath N., Jensen L., Stroux A., Böhm A. (2019). Immune system-mediated atherosclerosis caused by deficiency of long non-coding RNA MALAT1 in ApoE-/- mice. Cardiovasc. Res..

[109] Liang Z., Tang F. (2020). The potency of lncRNA MALAT1/miR-155/CTLA4 axis in altering Th1/Th2 balance of asthma. Biosci. Rep..

[110] Werner A. (2013). Biological functions of natural antisense transcripts. BMC Biol..

[111] Morrissy A.S., Griffith M., Marra M.A. (2011). Extensive relationship between antisense transcription and alternative splicing in the human genome. Genome Res..

[112] Rong J., Yin J., Su Z. (2015). Natural antisense RNAs are involved in the regulation of CD45 expression in autoimmune diseases. Lupus.

[113] Zhang T., Dong Z., Cai H., Rong J., Su Z. (2020). Estradiol regulates the expression of CD45 splicing isoforms in lymphocytes. Mol. Biol. Rep..

[114] Dvinge H., Guenthoer J., Porter P.L., Bradley R.K. (2019). RNA components of the spliceosome regulate tissue- and cancer-specific alternative splicing. Genome Res..

[115] Pu M., Chen J., Tao Z., Miao L., Qi X., Wang Y., Ren J. (2019). Regulatory network of miRNA on its target: Coordination between transcriptional and post-transcriptional regulation of gene expression. Cell. Mol. Life Sci..

[116] Gordon D.E., Jang G.M., Bouhaddou M., Xu J., Obernier K., White K.M., O’Meara M.J., Rezelj V.V., Guo J.Z., Swaney D.L. (2020). A SARS-CoV-2 protein interaction map reveals targets for drug repurposing. Nature.

[117] Srivastava R., Daulatabad S.V., Srivastava M., Janga S.C. (2020). Role of SARS-CoV-2 in Altering the RNA-Binding protein and miRNA-Directed Post-Transcriptional regulatory networks in humans. Int. J. Mol. Sci..

[118] Ashwal-Fluss R., Meyer M., Pamudurti N.R., Ivanov A., Bartok O., Hanan M., Evantal N., Memczak S., Rajewsky N., Kadener S. (2014). circRNA biogenesis competes with pre-mRNA splicing. Mol. Cell.

[119] Wilusz J.E. (2018). A 360 degrees view of circular RNAs: From biogenesis to functions. Wiley Interdiscip. Rev. RNA.

[120] Chen L.L. (2020). The expanding regulatory mechanisms and cellular functions of circular RNAs. Nat. Rev. Mol. Cell Biol..

[121] Zhu P., Zhu X., Wu J., He L., Lu T., Wang Y., Liu B., Ye B., Sun L., Fan D. (2019). IL-13 secreted by ILC2s promotes the self-renewal of intestinal stem cells through circular RNA circPan3. Nat. Immunol..

[122] Wang Y.H., Yu X.H., Luo S.S., Han H. (2015). Comprehensive circular RNA profiling reveals that circular RNA100783 is involved in chronic CD28-associated CD8(+)T cell ageing. Immun. Ageing.

[123] Li X., Liu C.X., Xue W., Zhang Y., Jiang S., Yin Q.F., Wei J., Yao R.-W., Yang L., Chen L.-L. (2017). Coordinated circRNA Biogenesis and Function with NF90/NF110 in Viral Infection. Mol. Cell.

[124] Liu C.X., Li X., Nan F., Jiang S., Gao X., Guo S.K., Xue W., Cui Y., Dong K., Ding H. (2019). Structure and degradation of circular RNAs regulate PKR activation in innate immunity. Cell.

[125] Aartsma-Rus A. (2010). Antisense-mediated modulation of splicing: Therapeutic implications for Duchenne muscular dystrophy. RNA Biol..

[126] MacKenzie A. (2012). Sense in antisense therapy for spinal muscular atrophy. N. Engl. J. Med..

[127] Arechavala-Gomeza V., Anthony K., Morgan J., Muntoni F. (2012). Antisense oligonucleotide-mediated exon skipping for Duchenne muscular dystrophy: Progress and challenges. Curr. Gene. Ther..

[128] Slansky J.E., Spellman P.T. (2019). Alternative splicing in tumors—A path to immunogenicity?. N. Engl. J. Med..

[129] Smith C.C., Selitsky S.R., Chai S., Armistead P.M., Vincent B.G., Serody J.S. (2019). Alternative tumour-specific antigens. Nat. Rev. Cancer.

[130] Shen S., Park J.W., Lu Z.X., Lin L., Henry M.D., Wu Y.N., Zhou Q., Xing Y. (2014). rMATS: Robust and flexible detection of differential alternative splicing from replicate RNA-Seq data. Proc. Natl. Acad. Sci. USA.

[131] Denti L., Rizzi R., Beretta S., Vedova G.D., Previtali M., Bonizzoni P. (2018). ASGAL: Aligning RNA-Seq data to a splicing graph to detect novel alternative splicing events. BMC Bioinform..

[132] Zhang Z., Zhou C., Tang L., Gong Y., Wei Z., Zhang G., Wang F., Liu Q., Yu J. (2020). ASNEO: Identification of personalized alternative splicing based neoantigens with RNA-seq. Aging.

[133] Oka M., Xu L., Suzuki T., Yoshikawa T., Sakamoto H., Uemura H., Yoshizawa A.C., Suzuki Y., Nakatsura T., Ishihama Y. (2021). Aberrant splicing isoforms detected by full-length transcriptome sequencing as transcripts of potential neoantigens in non-small cell lung cancer. Genome Biol..

[134] Garcia-Moreno J.F., Romao L. (2020). Perspective in alternative splicing coupled to nonsense-mediated mRNA decay. Int. J. Mol. Sci..

[135] Lu J., Plank T.D., Su F., Shi X., Liu C., Ji Y., Li S., Huynh A., Shi C., Zhu B. (2016). The nonsense-mediated RNA decay pathway is disrupted in inflammatory myofibroblastic tumors. J. Clin. Investig..

[136] Popp M.W., Maquat L.E. (2018). Nonsense-mediated mRNA decay and cancer. Curr. Opin. Genet. Dev..

[137] Perumal D., Imai N., Lagana A., Finnigan J., Melnekoff D., Leshchenko V.V., Solovyov A., Madduri D., Chari A., Cho H.J. (2020). Mutation-derived neoantigen-specific T-cell responses in multiple myeloma. Clin. Cancer Res..

[138] Mortezaee K. (2020). Immune escape: A critical hallmark in solid tumors. Life Sci..

[139] Hayashi H., Nakagawa K. (2020). Combination therapy with PD-1 or PD-L1 inhibitors for cancer. Int. J. Clin. Oncol..

[140] De Miguel M., Calvo E. (2020). Clinical challenges of immune checkpoint inhibitors. Cancer Cell.

[141] Rotival M., Quach H., Quintana-Murci L. (2019). Defining the genetic and evolutionary architecture of alternative splicing in response to infection. Nat. Commun..

